# Factors associated with excessive bleeding in cardiopulmonary bypass patients: a nested case-control study

**DOI:** 10.1186/1749-8090-2-17

**Published:** 2007-04-10

**Authors:** Juan J Jimenez Rivera, Jose L Iribarren, Jose M Raya, Ibrahim Nassar, Leonardo Lorente, Rosalia Perez, Maitane Brouard, Jose M Lorenzo, Pilar Garrido, Ysamar Barrios, Maribel Diaz, Blas Alarco, Rafael Martinez, Maria L Mora

**Affiliations:** 1 Intensive Care Unit, University Hospital of Canary Islands, La Laguna, Spain; 2 Hematology Department, University Hospital of Canary Islands, La Laguna, Spain; 3 Cardiac surgery Department, University Hospital of Canary Islands, La Laguna, Spain; 4 Research unit, University Hospital of Canary Islands, La Laguna, Spain; 5 Biochemistry and Central Laboratories, University Hospital of Canary Islands, La Laguna, Spain

## Abstract

**Introduction:**

Excessive bleeding (EB) after cardiopulmonary bypass (CPB) may lead to increased mortality, morbidity, transfusion requirements and re-intervention. Less than 50% of patients undergoing re-intervention exhibit surgical sources of bleeding. We studied clinical and genetic factors associated with EB.

**Methods:**

We performed a nested case-control study of 26 patients who did not receive antifibrinolytic prophylaxis. Variables were collected preoperatively, at intensive care unit (ICU) admission, at 4 and 24 hours post-CPB. EB was defined as 24-hour blood loss of >1 l post-CPB. Associations of EB with genetic, demographic, and clinical factors were analyzed, using SPSS-12.2 for statistical purposes.

**Results:**

EB incidence was 50%, associated with body mass index (BMI)< 26.4 (25–28) Kg/m^2^, (*P *= 0.03), lower preoperative levels of plasminogen activator inhibitor-1 (PAI-1) (*P *= 0.01), lower body temperature during CPB (*P *= 0.037) and at ICU admission (*P *= 0.029), and internal mammary artery graft (*P *= 0.03) in bypass surgery. We found a significant association between EB and 5G homozygotes for PAI-1, after adjusting for BMI (F = 6.07; *P *= 0.02) and temperature during CPB (F = 8.84; *P *= 0.007). EB patients showed higher consumption of complement, coagulation, fibrinolysis and hemoderivatives, with significantly lower leptin levels at all postoperative time points (*P *= 0.01, *P *< 0.01 and *P *< 0.01).

**Conclusion:**

Excessive postoperative bleeding in CPB patients was associated with demographics, particularly less pronounced BMI, and surgical factors together with serine protease activation.

## Background

When blood interacts with non-endothelial surfaces of the cardiopulmonary bypass machine, cellular and humoral pathways are activated, including the complement system, the coagulation system, and the fibrinolytic system. These in turn activate inflammatory response cells such as leukocytes and platelets. Together these molecular pathways and activated cells mediate the frequently observed clinical sequelae such as edema, tissue and organ damage, and hyperfibrinolysis [1]. Among these potential complications, bleeding is the most frequent and important. Postoperative hemorrhage is related to increased morbidity and mortality in patients undergoing cardiac surgery. Excessive postoperative bleeding has been attributed to acquired platelet dysfunction, impaired coagulation, and increased fibrinolysis. The characterization of the hemostatic defects responsible for EB is crucial for specific treatment and optimal clinical management of the patient [2], however, current risk stratification based on clinical, procedural, and biological markers is only partially successful and postoperative bleeding remains a serious problem for cardiac surgery patients. Major bleeding in cardiopulmonary bypass patients requires higher amounts of hemoderivatives and is associated with a higher incidence of reintervention, leading to a higher morbidity and mortality. When patients undergo reintervention, fewer than 50% exhibit surgical sources of bleeding [3].

Also important in the physiopathology of EB are specific host factors such as genetic predisposition to bleeding [4-6] or BMI [7], which may condition the organism's response to CPB and determine the equilibrium of the different systems activated by CPB.

The aim of this study was to analyze how activated serine proteases during CPB could be involved with excessive postoperative bleeding.

## Methods

### Design and study population

Postoperative care was performed in a polyvalent 24-bed intensive care unit (ICU), at a University hospital, Canary Islands (Spain). A total of 50 Caucasian patients undergoing elective CPB were considered for inclusion if they did not receive antifibrinolytic during or after the surgery, over a three-month period. Finally, we performed a nested case-control study of 26 patients who met the criteria. No patients had a history of hemostatic dysfunction, renal failure (Cr >2 mg/dL), chronic hepatopathy or immunosuppression. Before inclusion the participants had normal aggregation or coagulation functional test values (bleeding time, collagen/epinephrine and collagen/ADP closure time, prothrombin time, activated partial thromboplastin time and thrombin time). None of the patients received chronical immunosuppressive medications or other anti-inflammatory agents including acetyl salicylate acid or clopidogrel during the previous 5 days and the first 24 hours following intervention. This study was approved by our Hospital Ethics Committee. We obtained informed written consent from all patients for their inclusion in the study.

### Data collection

Demographic variables, comorbid conditions, perioperative clinical data and postoperative outcomes (duration of mechanical ventilation, Intensive Care Unit and hospital stay and mortality) were recorded. Core body temperature, biochemical determinations (hematology, coagulation, fibrinolysis and complement parameters) and hemodynamic parameters were recorded at four time points: before intervention, after surgery at 0 h and at 4 h and 24 hours post-intervention. In addition, we recorded blood loss measured by tube chest drainage and the amount of hemoderivatives used after intervention at the above time points and when chest tubes were removed (defined as total blood loss). Excessive bleeding was defined as blood loss >1 L over 24 hours after intervention. Surgical risk was calculated by parsonnet score and severity on admission by APACHE-II and SAPS II scores.

### Perioperative management

The same anesthetic procedure was used in all patients. All interventions were performed by the same surgical team. The extracorporeal circuit consisted of a hardshell membrane oxygenator (Optima XP, Cobe, Denver, Colorado or Quantum Lifestream International, Inc. TX USA), a Tygon™ extracorporeal circuit and a Medtronic™ Biopump centrifugal pump. Under 28–30°C hypothermia, the pump flow was adjusted to maintain a mean arterial pressure of >60 mmHg and a flow index of 2.2 l/min/m^2^. Myocardial protection was achieved using St. Thomas 4:1 sanguineous cardioplegia. The circuit was primed with 30 mg of heparin, followed by an initial dose of 3 mg/Kg and necessary doses to achieve and mantain an activated clotting time of 480 s. To reverse the effect of heparin, protamine was used based on blood heparin levels measured by Hetcon^® ^(HMS.Medtronic™).

### Leptin levels

Serum leptin levels (normal range: <50 ng/mL; intra-assay variation: 3%; inter-assay variation: 6.7%) were measured using Immunoradiometric Assay (IRMA) (Diagnostic Division Abbott).

### Complement determination

We recorded sequential levels of C3 (normal range 60–150 mg/dL; intra-assay variation: 1.2%; inter-assay variation: 1.4%), C4 (normal range: 10–50 mg/dL; intra-assay variation: 2%; inter-assay variation: 2.5%), C1q (normal range: 10–25 mg/dL; intra-assay variation: 2.3%; inter-assay variation: 3.4%), C1 inh (normal range: 15–33 mg/dL; intra-assay variation: 1.4%; inter-assay variation: 1.8%) and B Factor (normal range: 10–40 mg/dL; intra-assay variation: 2.2%; inter-assay variation: 3.2%) by nephelometry, (IMMAGE-Beckman Instruments. Inc. Fullerton, CA). C7 component (normal range: 4–10 mg/dL; intra-assay variation: 3%; inter-assay variation: 4.6%) was determined by radial immunodiffusion.

### Coagulation and fibrinolysis determination

Quantitative plasminogen activator inhibitor type-1 (PAI-1) antigen (normal range: 2–47 ng/mL; intra-assay variation: 3.7%; inter-assay variation: 4.3%) and tissue plasminogen activator (t-PA) antigen (normal range: <9.0 ng/mL; intra-assay variation: 4.2%; inter-assay variation: 4.0%) were measured using ELISA test (IMUBIND^®^, America Diagnostica Inc., Stanford, Connecticut, USA). D-dimer (normal range: <300 ng/mL; intra-assay variation: 3%; inter-assay variation: 3.8%) was measured using immunoturbidimetric test (D-Dimer PLUS, Dade Behring, Marburg, Germany).

### Gene analysis

Blood samples (3 ml) were collected before surgery in EDTA-containing tubes and subjected to DNA purification using proteinase K, phenol-chloroform extraction, and ethanol precipitation. Genotyping was performed in a blinded manner, without knowledge of any clinical data. Gene analysis of PAI-1G4/G5 polymorphisms was performed in all patients using primers and restriction endonuclease digestion. In addition, 22 neutral markers were genotyped to follow genomic control strategies that would detect spurious associations due to population substructure. The neutral markers chosen were Alu repeats distributed throughout the genome.

### Statistical analysis

Pearson's chi^2 ^and Fisher exact test were used, Student t test for independent groups and Mann-Whitney U test for non-parametric variables. We compared the ranges of PAI-1 levels between the different genotypes of PAI-1 polymorphism using Jonckheere-Terpstra test. Statistical significance was defined as a *P*-value of less than 0.05. Data are expressed as median values and interquartile range. Logistic regression was used for calculating odds ratios and 95 percent confidence interval between preoperative PAI-1 levels and excessive bleeding, controlling for BMI and temperature. Blood loss at 24 hours was log-transformed and an analysis of covariance (ANCOVA) was used to compare genotypes (5G/5G vs 4G/5G and 4G/4G, of PAI-1 polymorphisms), controlling for BMI and temperature in two independent models.

## Results

**Figure 1:**
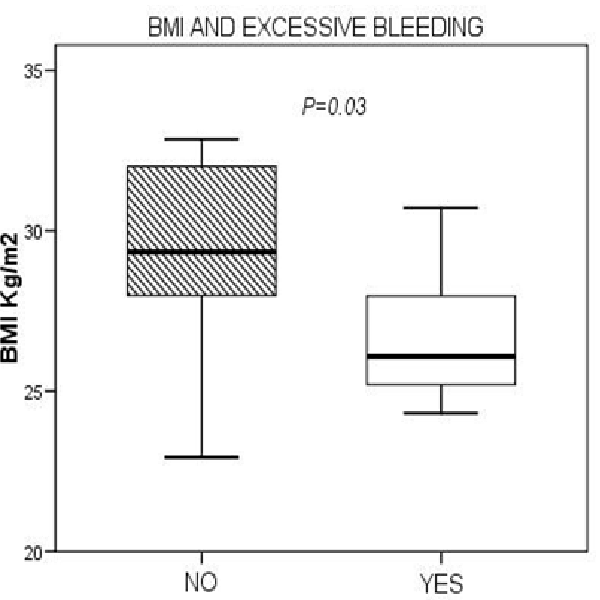
Horizontal line represents the median, box encompasses the 25^th^–75^th ^percentile, and error bars encompass the 10^th^–90^th ^percentile.

EB was observed in 13 out of 26 patients (50%). We found statistically significant associations between EB and patients in the lower BMI group <26.4 (25–28) Kg/m^2 ^(*P *= 0.03) (Figure 1). Lower preoperative levels of PAI-1 (*P *= 0.014) were associated with EB. Regarding surgical procedure, EB was significantly greater in those patients who received internal mammary artery graft (*P *= 0.03) in bypass surgery, and in those who had lower temperature during cardiopulmonary bypass (*P *= 0.04).

**Figure 2:**
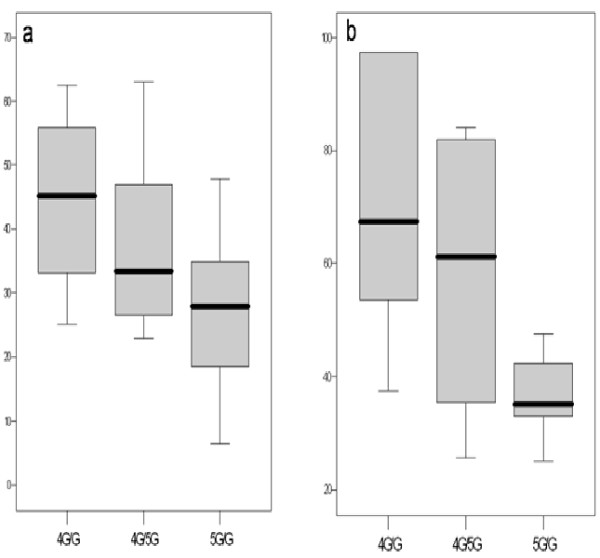
PAI-1 levels according to PAI-1genotypes. A. Preoperative. B. ICU admission (0-hr).

The distribution of PAI-1 polymorphism was 4G/G in 5 patients (19%), 4G/5G in 12 (46%), and 5G/G in 9 (35%), similar to that observed in 100 consecutive blood donors from our institution (4G/G: 21%; 4G/5G: 52%; 5G/G: 27%) and was in Hardy Weinberg equilibrium. With respect to postoperative EB according to PAI-1 polymorphism, we observed that 1 out of 5 (20%) of 4G/G genotype, 5 out of 12 (42%) of 4G/5G and 7 out of 9 (78%) of 5G/G genotype, showed postoperative EB. Using analysis of covariance (ANCOVA), EB was significantly associated with the 5G homozygote for the PAI-1 polymorphism after adjusting for BMI (*P *= 0.02) and temperature during CPB (*P *= 0.007). The three genotypes of PAI-1 polymorphism showed significant differences in preoperative (Fig 2a) and 0h (Fig 2b) PAI-1 levels, (*P *= 0.046 and *P *= 0.018) respectively. 5G/5G patients presented 24-hour blood loss of 1190 cc (979 cc–1763 cc) whereas 4G/4G and 4G/5G patients presented 490 cc (365 cc–605 cc) and 715 cc (475 cc–1175 cc), respectively (*P *= 0.021).

EB patients had significant higher consumption of several components of complement at 0 hours: C1q (*P *= 0.02), C1-inhibitor (*P *= 0.03), B Factor (*P *= 0.03) and C7 (*P *< 0.01). At 4 hours these patients had significant lower levels of C1q (*P *< 0.01), C1-inhibitor (*P *= 0.04), C3 (*P *= 0.01), B Factor (*P *= 0.02) and C7 (*P *< 0.01).

With respect to coagulation, patients with EB presented significantly lower levels of prothrombin time (PT) (*P *= 0.04) at 0 hours, as well as lower levels of PT (*P *= 0.03) and fibrinogen (*P *= 0.03) at 4 hours. Regarding fibrinolysis, we did not find differences in t-PA levels between groups. However, EB patients showed lower levels of PAI-1 (*P *< 0.01) and lower PAI-1/t-PA ratio (*P *= 0.014) at 0 hours, with higher levels of D-dimer at 4 hours 1015 (944–1177) ng/mL vs 895 (753–905) ng/mL in the non-EB group (*P *= 0.18). Serum leptin levels were significantly lower at all postoperative time points (*P *= 0.01, *P *< 0.01 and *P *< 0.01) in the EB group, which remained significant after adjusting for BMI (F: 17.5; *P *= 0.001). They also showed significantly lower hematocrit levels at 4 h (*P *= 0.048) and lastly, greater transfusion requirements: total red blood cells (*P *< 0.01) and total plasma (*P *= 0.047). See Table 1.

**Table 1:** Characteristics of patients with excessive bleeding vs those without^a^

**Variables**	**< 1000 cc (n = 13)**	**> 1000 cc (n = 13)**	**P**
**Demographic**			
Sex (% males)	6 (46)	9 (69)	*0.21*
Age (years)	69 (60–75)	64 (61–75)	*0.88*
Body Mass Index (BMI) (kg/m^2^)	29.3 (28–32)	26.1 (25–27.9)	*0.03*
APACHE-II	13 (11–16)	14 (12–18)	*0.21*
SAPS-II	31 (31–32)	31.5 (29–40)	*0.78*
Parsonnet	17.5 (13.5–21)	15(9–20.5)	*0.55*
**Surgical parameters**			
Type of bypass (%)			*0.03*
Venous	4 (31)	0	
Arterial and venous	8 (61.5)	4 (31)	
Cardiopulmonary bypass time (min)	85 (77–110)	100 (67–110)	*0.84*
Aortic clamping time (min)	63 (45–71)	55 (50–70)	*0.96*
Temperature during CPB (°C)	32 (31.5–32)	31 (30–32)	*0.04*
**Laboratory parameters**			
C1q 0 h (mg/ml)	10.4(8.7–16.5)	6.9 (6.2–9.1)	*0.02*
C1q 4 h (mg/ml)	14.9 (9.3–16)	7.6 (7.1–9.8)	*<0.01*
C1q 24 h (mg/ml)	12.8 (9.9–15.6)	8.4 (7.2–10.1)	*0.04*
C1-Inhibitor 0 h (mg/ml)	20.4 (16.6–21.4)	13.2 (10.4–16.8)	*0.03*
C1-Inhibitor 4 h (mg/ml)	19.5 (16.2–20.5)	13.2 (10.8–17)	*0.04*
C4 24 h (mg/ml)	21.2 (17–22.5)	14.7 (13.8–18.8)	*0.04*
C3 4 h (mg/ml)	87 (76–94)	85(70–88)	*0.01*
B Factor 0 h (mg/ml)	21.4 (18.9–26.4)	15.6 (15.3–20.2)	*0.03*
B Factor 4 h (mg/ml)	24.7 (18.7–28.2)	18.6 (17–19.7)	*0.02*
C7 0 h (mg/ml)	6.3(5.4–8.5)	3.3 (2.5–5.9)	*<0.01*
C7 4 h (mg/ml)	6.6 (5–9.6)	3.3 (2.1–4.6)	*<0.01*
Leptin 0 h (ng/ml)	12.1 (7.5–22.4)	5.5 (1.9–7.7)	*0.01*
Leptin 4 h (ng/ml)	10.7 (7.8–19.2)	4.2 (1.4–6.2)	*<0.01*
Leptin 24 h (ng/ml)	32.6 (20.4–48.1)	6.9 (3.8–12.3)	*<0.01*
Prothrombin time admission (%)	77 (72–79)	70 (56–71)	*0.04*
Prothrombin time 4 h (%)	77 (68–81)	67 (64–69)	*0.03*
Fibrinogen 4 h (mg/ml)	351 (272–409)	272 (209–319)	*0.03*
Plasminogen activator inhibitor-1 preoperative (ng/ml)	43.5 (31.5–52.8)	25.5 (23–36)	*0.01*
Plasminogen activator inhibitor-1 0 h (ng/ml)	76.6(53.5–97.3)	35.5 (32.1–42.6)	*<0.01*
PAI-1/t-PA ratio 0 h	3.7 (2.7–6.8)	1.6 (1.2–3.4)	*0.014*
Hematocrit (%) 4 h	28.6 (25.8–31.9)	25.4 (24–27.8)	*0.048*
Total red blood cells (ml)	400 (0–800)	1600 (800–2000)	*<0.01*
Total plasma frozen (ml)	0	800 (0–900)	*0.047*
Blood loss 4 h (mL)	661 (572–992)	220 (170–520)	*0.01*
Blood loss 24 h (mL)	1190 (978–1762)	605 (470–1100)	*0.01*

^a^Values are expressed as median and ranges.

## Discussion

In patients who receive prophylaxis against bleeding, the frequency of excessive bleeding after CPB is around 30%, depending on the definition used and surgical procedure [8]. Excessive perioperative bleeding continues to complicate CPB surgery and in some hospitals more than 25% of all blood products are assigned to open heart surgery [9]. We observed excessive blood loss (>1 L/24 h) in 50% of the patients after elective cardiac surgery under CPB, none of whom received antifibrinolytic prophylaxis.

Even when neutral markers partially guarantee genetics associations, the relationship between 5G homozygosity of PAI-1 polymorphism and EB must be cautiously considered given the small sample size.

The PAI-1 4G/5G polymorphism is known to influence plasma levels of PAI-1, the main regulator of fibrinolysis, the 4G allele being associated with high levels and the 5G allele with low levels [10]. Much attention has focused on the 4G/4G polymorphism; however, there are very few reports in the literature of the importance of the 5G/5G polymorphism. In our study, homozygous patients for 5G presented lower PAI-1 levels preoperatively and at ICU admission, as well as greater blood loss than other genotypes. Besides, lower preoperative levels of PAI-1 and lower ratio PAI-1/t-PA just after surgery might lead to higher levels of D-dimer later on: this indicates that there are patients with a lower inhibitory potential for fibrinolysis prior and immediately after surgery, possibly favoring EB. These patients may benefit from the use of antifibrinolytic prophylaxis [11,12].

Within a range of slight overweight, the patients in the lower BMI group showed EB, a finding which agrees with those of recently published reports, although the strong relationship between BMI and excessive bleeding remains unexplained [7,13]. Obesity is associated with increased blood concentrations of endothelial dysfunction markers such as fibrinogen and Von Willebrand factor (vWF) [14], associated with hypercoagulability [15]. Obesity and insulin resistance seem to be the most important factors influencing the level of PAI-1 [16], and on the other hand the PAI-1 gene polymorphism is known to play a determinant role in the regulation of PAI-1 plasma levels in the obese, acting at the adipose tissue level [17]. Moreover a correlation between PAI-1 and leptin has recently been reported in humans [18]. In our study, leptin levels were associated with EB. Leptin is the 167-amino acid protein product of the obese (ob) gene, which is secreted from adipocytes. Leptin receptors are widely expressed, raising the possibility that leptin may have broad effects. In fact, it is now clear that leptin influences a variety of physiological and pathological processes including angiogenesis and vascular disorders. In this regard, leptin-deficient ob/ob mice, and wild-type mice treated with a leptin-neutralizing antibody, develop unstable thrombi and an attenuated thrombotic response to arterial injury, and they exhibit a defect in platelet aggregation [19,20]. This underlines the importance of genetic background when assessing the reaction of an organism to insults, such as CPB in this case. Nevertheless, data on potential interactions between environmental and metabolic variables on one hand, and the genetic predisposition on the other, are still scarce.

Lower core body temperature during CPB and the use of internal mammary artery graft in cardiac surgery have been described as risk factors for postoperative bleeding [7,21].

Excessive bleeding after cardiac surgery is generally related to a combination of several factors associated with CBP. Activation of the coagulation cascade gives rise to thrombin formation which in turn activates the inflammatory system, including complement [22]. Activation of fibrinolysis occurs simultaneously, enhanced by complement [23]. Complement activation has been associated with postoperative bleeding and tissue injury [24,25]. This activation occurs during CPB and after neutralization of heparin with protamine [26]. In a recent study, fifteen minutes after heparin reversal, patients were at risk for excessive bleeding, when enhanced fibrinolysis was observed [27].

In our study all these alterations were observed in the patients with excessive postoperative bleeding; at ICU admission (0 hours) some 20 minutes after heparin reversal, they showed greater consumption of coagulation, complement and leptin, together with increased fibrinolysis, as compared to those without EB. These alterations persisted during the first 4 hours after surgery when the greatest amount of blood loss was recorded. Consequently, EB patients had greater transfusion requirements.

Taking into account the complexity of excessive bleeding in cardiac surgery with extracorporeal circulation, it is difficult to establish the relative contribution of each of these components on the development of this important clinical picture. We consider that the main limitation of the study is the small sample size, and the findings require confirmation in larger series.

## Conclusion

Excessive postoperative bleeding in CPB patients was associated with demographics, particularly less pronounced BMI, and surgical factors together with serine protease activation.

## Abbreviations

EB: excessive bleeding.

CPB: cardiopulmonary bypass.

BMI: body mass index.

PAI-1: plasminogen activator inhibitor-1.

IMA: internal mammary artery.

t-PA: tissue plasminogen activator

PT: prothrombin time.

APTT: activated partial thromboplastin time.

## Competing interests

The author(s) declare that they have no competing interests.

## Authors' contributions

JJJ and JLI conceived of the study and participated in its design and coordination, as well as in the statistical analysis and data interpretation.

LL, RP, MB, JML and MLM carried out data collection, processing blood simples during the study and useful suggestions.

JMR carried out the coagulation and fibrinolysis immunoassays and interpretation.

IN, PG and RM were the surgical team and carried out the preoperative clinical and analytical data collection.

YB performed the molecular genetic studies.

BA and MD carried out the determination of complement, leptins, soluble TNF receptors, IL-6 and interpretation.

All authors read and approved the final manuscript.
